# Genetic analyses of the bidirectional associations between common mental disorders and asthma

**DOI:** 10.3389/fpsyt.2024.1372842

**Published:** 2024-06-06

**Authors:** Zian Yan, Jingjing Chen, Lijun Guo, Hongwei Zhang, Yanqiu Ding, Gaocan Ren, Yiyi Mao, Ruina Bai, Xiaochang Ma

**Affiliations:** ^1^ Graduate School, Beijing University of Chinese Medicine, Beijing, China; ^2^ Xiyuan Hospital, China Academy of Chinese Medical Sciences, Beijing, China

**Keywords:** mendelian randomization, causality, asthma, mental disorder, anxiety, depression

## Abstract

**Objective:**

Although extensive research has explored the link between mental disorders and asthma, the characteristics and patterns of this association are still unclear. Our study aims to examine the genetic causal links between common mental disorders (specifically, anxiety and depression) and asthma.

**Methods:**

We conducted genetic analyses including linkage disequilibrium score regression (LDSC) and bidirectional two-sample Mendelian randomization (MR) analyses, and utilized summary statistics from recent large-scale Genome-Wide Association Studies (GWASs) in European populations, covering sensation of anxiety or depression, anxiety sensation, depression sensation, anxiety disorders, major depression disorder (MDD), and asthma.

**Results:**

LDSC revealed significant genetic correlations among sensation of anxiety or depression, MDD and asthma (*P* < 0.017), highlighting potential genetic correlation between anxiety disorders and asthma (*P* < 0.05 yet > 0.017). In bidirectional two-sample MR, inverse-variance weighted (IVW) analyses suggested that genetic liability to asthma was significantly associated with an increased risk of sensation of anxiety or depression (OR = 4.760, 95%CI: 1.645–13.777), and MDD (OR = 1.658, 95%CI: 1.477–1.860). Conversely, IVW analyses indicated that genetic liability to anxiety disorders was not associated with an increased risk of asthma (*P* > 0.01), nor was genetic liability to asthma associated with an increased risk of anxiety disorders (*P* > 0.01). Furthermore, no significant genetic causal relationships were observed for other studied traits. Multivariate MR, after adjusting for body mass index and alcohol consumption, further corroborated the independent causal effect of genetic predisposition to MDD on the risk of asthma (OR = 1.460, 95% CI: 1.285–1.660).

**Conclusion:**

Our study establishes MDD as a predisposing factor for asthma. Meanwhile, anxiety disorders are not causal risk factors for asthma, nor is the reverse true. It is recommended to closely monitor asthma symptoms in patients with MDD.

## 1 Introduction

Anxiety and depression, common mental disorders, have significant disability rates (1). Despite the frequent comorbidity of anxiety and depressive disorders (2), they exhibit distinct symptoms. Common symptoms of anxiety include fear, nervousness, and worry (3). Approximately 34% of American adults experience lifelong anxiety (4). Depression, characterized by emotional downturns, social impairments, and motor retardation, significantly limits psychosocial functioning and life quality (5).

Asthma, a chronic non-infectious respiratory condition, is marked by tracheobronchial obstruction and airway hyperresponsiveness (6). The 2015 Global Burden of Disease Study reports that asthma affects 358 million people globally, up 12.6% from 1990, highlighting its emergence as a major public health issue (7). Research shows that 18.1% of adults with asthma also suffer from depression (8). Given the aging demographic, the prevalence of asthma and associated mental health issues is likely to increase (9). Thus, understanding the intricate relationship between anxiety, depression, and asthma is crucial for both disease prevention and complication management.

Observational studies have explored the anxiety, depression, and asthma nexus, but clear patterns remain elusive. An international survey (10) found higher odds ratio (OR) of mental disorders in asthma patients: 1.6 for depression and 1.5 for anxiety (95% CIs: 1.4–1.8 and 1.4–1.7, respectively). Lehto et al. (11) reported associations between asthma and affective traits, with ORs of 1.67 for major depression and 1.45 for anxiety (95% CIs: 1.50–1.86 and 1.30–1.61). A meta-analysis (12) showed higher risks of anxiety symptoms or anxiety disorders in asthma patients (ORs: 1.89 and 2.08, 95% CIs: 1.42–2.52 and 1.70–2.56). These findings highlight the close relationship between asthma and psychological disorder and suggest that mental symptoms and mental disorders are also differentially related to asthma. However, the causal links, especially genetic ones, between anxiety, depression, and asthma are unclear, highlighting the need for more comprehensive, bidirectional studies.

In addition, conventional observational studies often struggle to infer causality accurately due to biases from unmeasured confounding factors and reverse causality. Mendelian randomization (MR) offers an alternative approach to measure causal effects more reliably (13). MR addresses these challenges effectively by leveraging the random genetic assignment during gametogenesis (14). By employing genetically determined variants as instrumental variables (IVs), MR discerns causal links between exposures and outcomes, minimizing risks of confounding and reverse causality (15).

**Figure 1:**
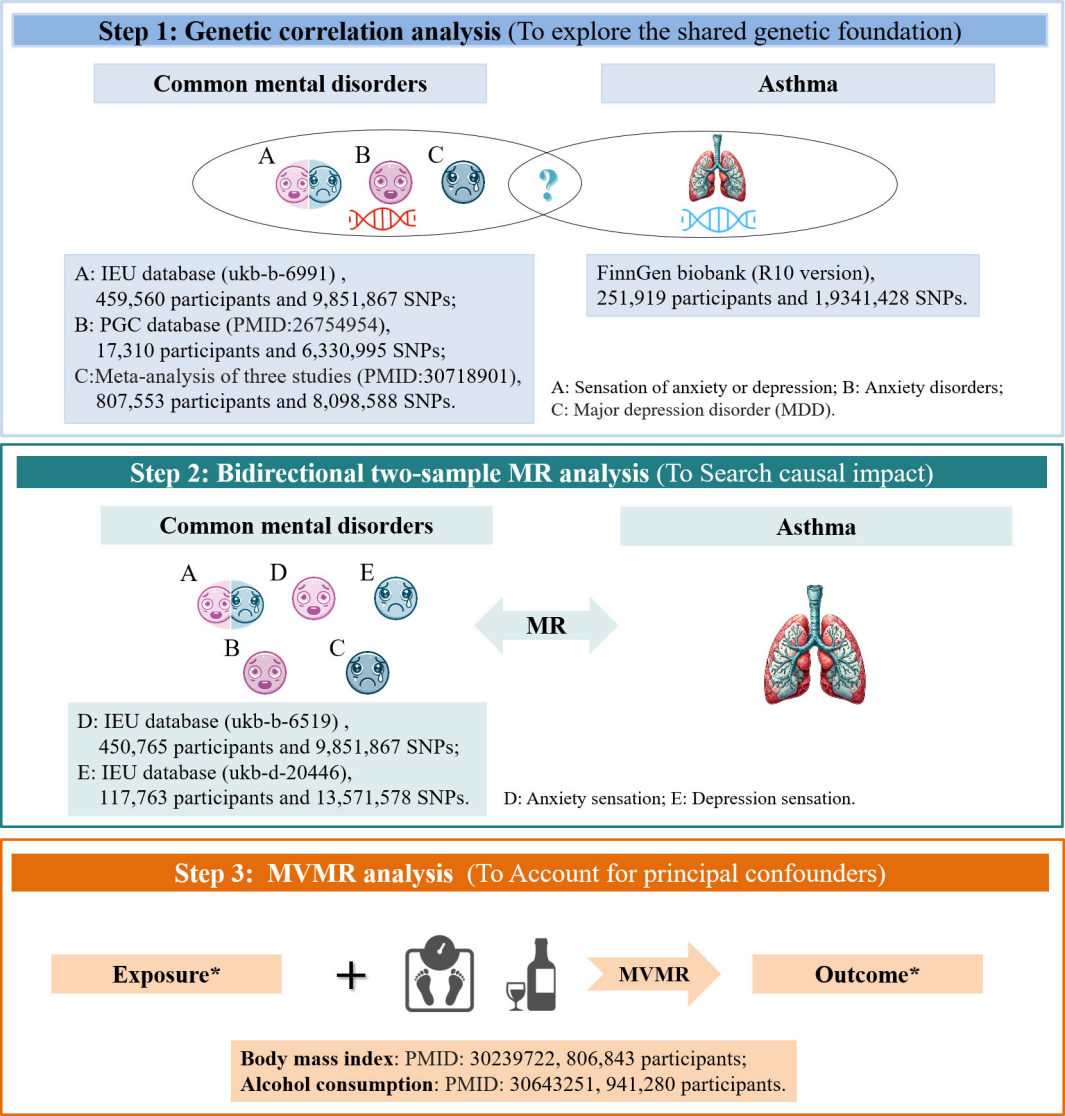
Study Design and Data Source. *In cases where univariable MR indicated a significant causal link. MR, Mendelian randomization; MVMR, multivariable MR; PCG, Psychiatric Genomics Consortium.

Thus, to investigate the causal nature of this association, we utilized summary statistics from extensive genome-wide association studies (GWASs) and conducted a complexed genetic study comprising: (i) a genetic correlation analysis to determine the shared genetic foundation among anxiety, depression, and asthma; (ii) a bidirectional two-sample MR analysis to explore the causal impact of these relevant traits; and (iii) an inverse-variance weighted (IVW) multivariate MR (MVMR) analysis to account for principal confounders in univariable MR that revealed significant causal connections, as shown in **Figure 1**. By integrating genetic evidence, our aim was to clarify the nature of the previously established link between anxiety, depression, and asthma. This understanding may offer a scientific foundation for the prevention and treatment strategies of these conditions.

## 2 Materials and methods

### 2.1 Data source

Utilizing the IEU database (https://gwas.mrcieu.ac.uk), we gathered GWAS datasets for anxiety and depression, identifying results for sensation states (labeled as “sensation of anxiety or depression”, “anxiety sensation”, and “depression sensation”) and clinical diagnoses (“anxiety disorders” and “major depression disorder”, MDD). This approach enabled us to stratify data based on disease severity, resulting in a hierarchical and progressive categorization of the outcomes. A fundamental aspect of two-sample MR is ensuring minimal or no sample overlap between exposure and outcome data, within acceptable limits (16). Consequently, we chose the latest R10 version of the GWAS datasets for asthma from the FinnGen database (17), released in December 2023.

The GWAS datasets utilized in this study entirely comprise European population samples. Detailed information is provided in **Figure 1** and **Supplementary Table S1**.

No additional ethical approval was needed as all data are publicly accessible.

### 2.2 IVs selection

As for MR, we meticulously chose IVs consisting of independent, genome-wide significant SNPs from exposure and outcome datasets, forming a robust foundation for our MR analysis. To fulfill the three fundamental hypotheses of MR (18), we selected SNPs demonstrating strong correlation with exposure (*P* < 5×10–^8^) as instrumental variables for analyzing disease causality. To prevent linkage disequilibrium bias, significant SNPs linked to exposure factors must meet these criteria: r^2^<0.001 and a genetic distance exceeding 10,000kb. SNPs that don’t meet the independence criteria from confounding factors are excluded. We extracted SNPs related to exposure factors from the GWAS dataset, excluding those directly linked to the outcome (*P* < 5×10–^5^) to assure SNPs influence the outcome solely via exposure. To ensure robust results, the exclusion threshold for the Steiger Test has been set to a more stringent level of *P* < 5×10–^5^. Ultimately, we coordinated the data ensuring that the effects of SNPs on exposure and outcome were associated with the same allele. In analyzing “depression sensation” and “anxiety disorders” as exposure factors, we applied a less stringent threshold of *P* < 5×10–^6^ (19).

Upon finalizing the IVs for analysis, we record key data including allelic effect values (β), standard errors (SE), effect allele frequency (eaf), and *P*-values. We assessed the strength of the IVs using the F statistic, which is a function of the magnitude and precision of the genetic effect on the trait: F = R^2^ ×(N - 2)/(1 - R^2^), where R^2 =^ 2 × eaf × (1 - eaf) × β^2^, and N is the sample size of the GWAS of SNPs with the trait. We only consider SNPs with an F-value greater than 10 as robust, as this indicates strength against weak instrument bias; those with lower F-values are rejected.

### 2.3 Genetic correlation analysis

Linkage disequilibrium score regression (LDSC) has been utilized to assess the overall genetic correlation between “sensation of anxiety or depression”, “anxiety disorders”, “MDD”, and asthma (20). LDSC, grounded in genetic linkage disequilibrium (LD) principles, evaluates the genetic contributions to complex diseases and traits by quantifying LD associations between SNPs and their neighboring SNPs. Genome-wide SNP analysis reveals more about genetic etiologies than MR with selected SNPs (21). Genetic correlation estimates (r_g_) vary from -1 to +1, where -1 represents a perfect negative correlation, +1 a perfect positive correlation, and values closer to -1 or +1 signify stronger correlations.

### 2.4 Mendelian randomization analysis

We used a bidirectional two-sample MR approach to investigate the genetic causality between anxiety, depression, and asthma. The analysis entailed two steps: first, a forward MR with asthma as the outcome, followed by a reverse MR using asthma as the exposure. Sensitivity analysis using MR Egger and IVW methods validated the results.

Analyses encompassed bidirectional MR via five methods. IVW was the primary method in the absence of horizontal pleiotropy, alongside weighted median, MR-Egger regression, simple mode, and weighted mode. IVW aggregated SNP MR effect estimates for a comprehensive causal effect estimate (22), which was most reliable without horizontal pleiotropy (23) and transformed the data into OR and 95% confidence intervals (CI). MR-PRESSO identifies and corrects horizontal pleiotropy, eliminating outliers (24). Persistent pleiotropy leads to further outlier removal via RadialMR (25). IVW and MR-Egger regression quantified heterogeneity. A leave-one-out sensitivity analysis was conducted, sequentially omitting each SNP to assess its impact on the results.

In cases where univariable MR indicated a significant causal link between mental disorders and asthma, we conducted an IVW-based MVMR analysis (26) to adjust for major confounders including body mass index (BMI) and alcohol consumption (27). Summary-level association results were derived from the largest available GWAS for each phenotype, involving 806,843 (28) and 941,280 (29) individuals respectively. In the presence of heterogeneity in IVW results, the weighted median and MR-Egger methods are employed to yield a more robust causal inference (30).

### 2.5 Statistical analysis

Analyses utilized “TwoSampleMR” and “MR-PRESSO” in R Software (version 4.3.2) (31, 32). For multiple testing in LDSC, a Bonferroni-adjusted significance threshold was set at *P* < 0.017 (derived from 0.05/3) (33). Similarly, for multiple testing in two-sample MR, the Bonferroni correction was applied to set the significance threshold at *P* < 0.01 (0.05/5) (34) as well. *P*-values below 0.05 yet above the Bonferroni threshold suggest potential associations. In determining causality, a *P*-value < 0.05 combined with an OR > 1 implies positive genetic causality; conversely, an OR < 1 suggests negative genetic causality. A *P*-value of < 0.05 was considered statistically significant for MVMR. In MR-PRESSO, a *P*-valued ≥ 0.05 implies no pleiotropy. In IVW and MR-Egger, a *P*-value ≥ 0.05 suggests the absence of heterogeneity.

## 3 Results

### 3.1 Genetic correlation analysis

The results of the genetic correlation analyses between overall sensation (sensation of anxiety or depression), two mental disorders (anxiety disorders and MDD), and asthma are shown in **Table 1**. A significant positive genetic correlation was observed between sensation of anxiety or depression, MDD and asthma (*P* < 0.017). Additionally, a potential genetic correlation between anxiety disorders and asthma was noted (*P* < 0.05 yet > 0.017). These findings suggest a shared genetic basis for these complex phenotypes, including those with a potential correlation.

**Table 1:** Genetic correlation between mental disorders and asthma.

Trait 1	Trait 2	r_g_	SE	*P*
Sensation of anxiety or depression	Asthma	0.354	0.037	1.25E-21
Anxiety disorders		0.300	0.128	0.019
Major depression disorder		0.407	0.035	1.01E-30

r_g_, genetic correlation estimate; SE, standard error.

### 3.2 The causal effect of anxiety and depression on asthma (forward MR)

**Figure 2:**
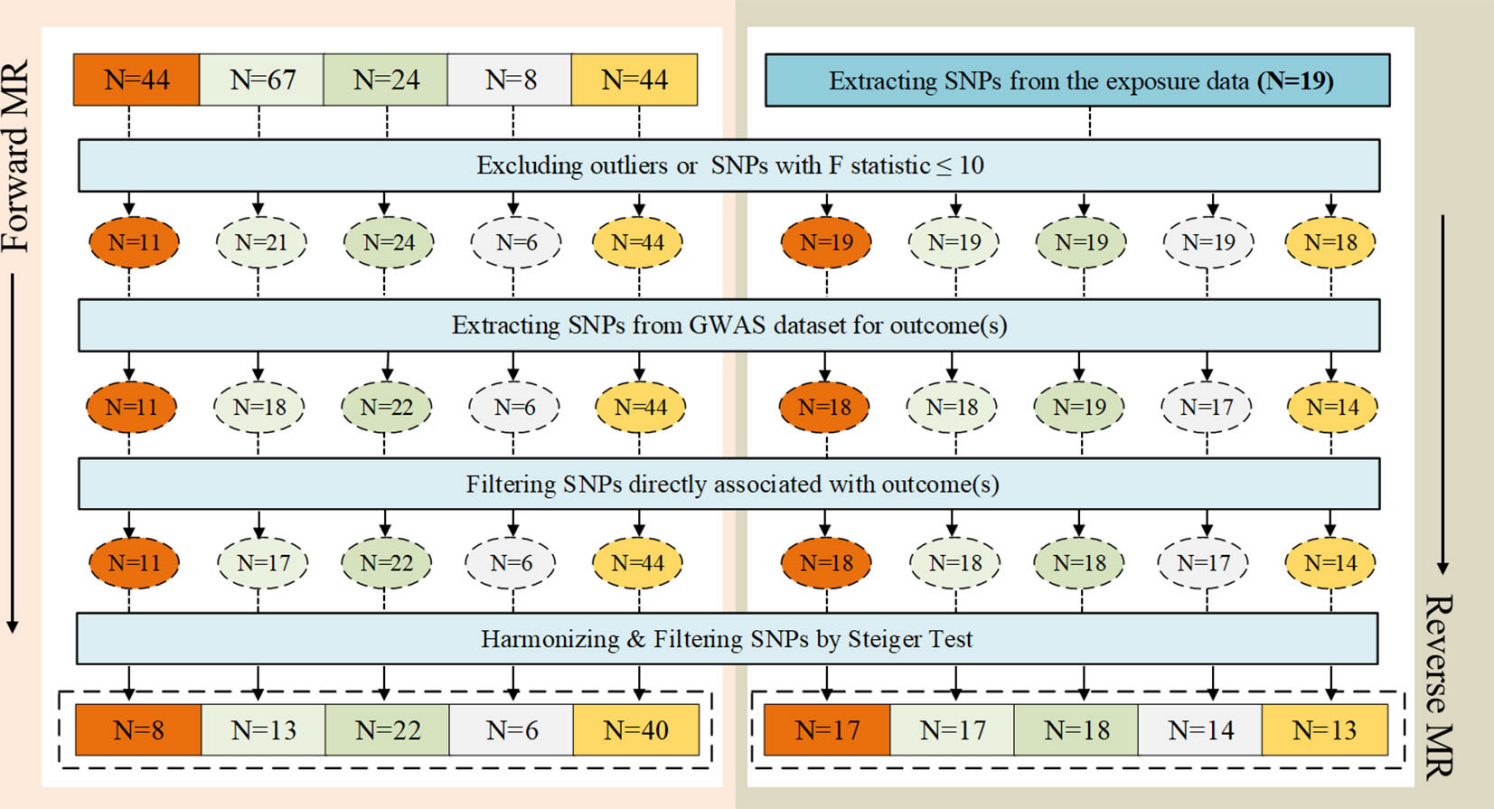
The selection process of instrumental variables (IVs). **(A)** Sensation of anxiety or depression, **(B)** Anxiety sensation, **(C)** Depression sensation, **(D)** Anxiety disorders, **(E)** Major depression disorder (MDD). A 
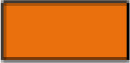
 B 
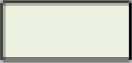
 C 
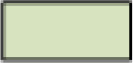
 D 
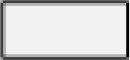
 E 
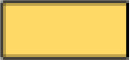

Considering the correlation between overall sensation (sensation of anxiety or depression) and asthma, anxiety sensation and depression sensation were included in the following bidirectional two-sample MR analysis, along with the three traits mentioned above. From the exposure data, we extracted 44, 67, 24, 8, and 44 SNPs, respectively. Ultimately, 8, 13, 22, 6, and 40 SNPs were utilized, each with an F statistic > 10, as illustrated in **Figure 2**. After excluding outliers for two exposure variables (“anxiety sensation”, “MDD”), no evidence of pleiotropy was observed in the subsequent pleiotropy test. Therefore, MR analysis was performed using IVW as the primary method.

**Figure 3:**
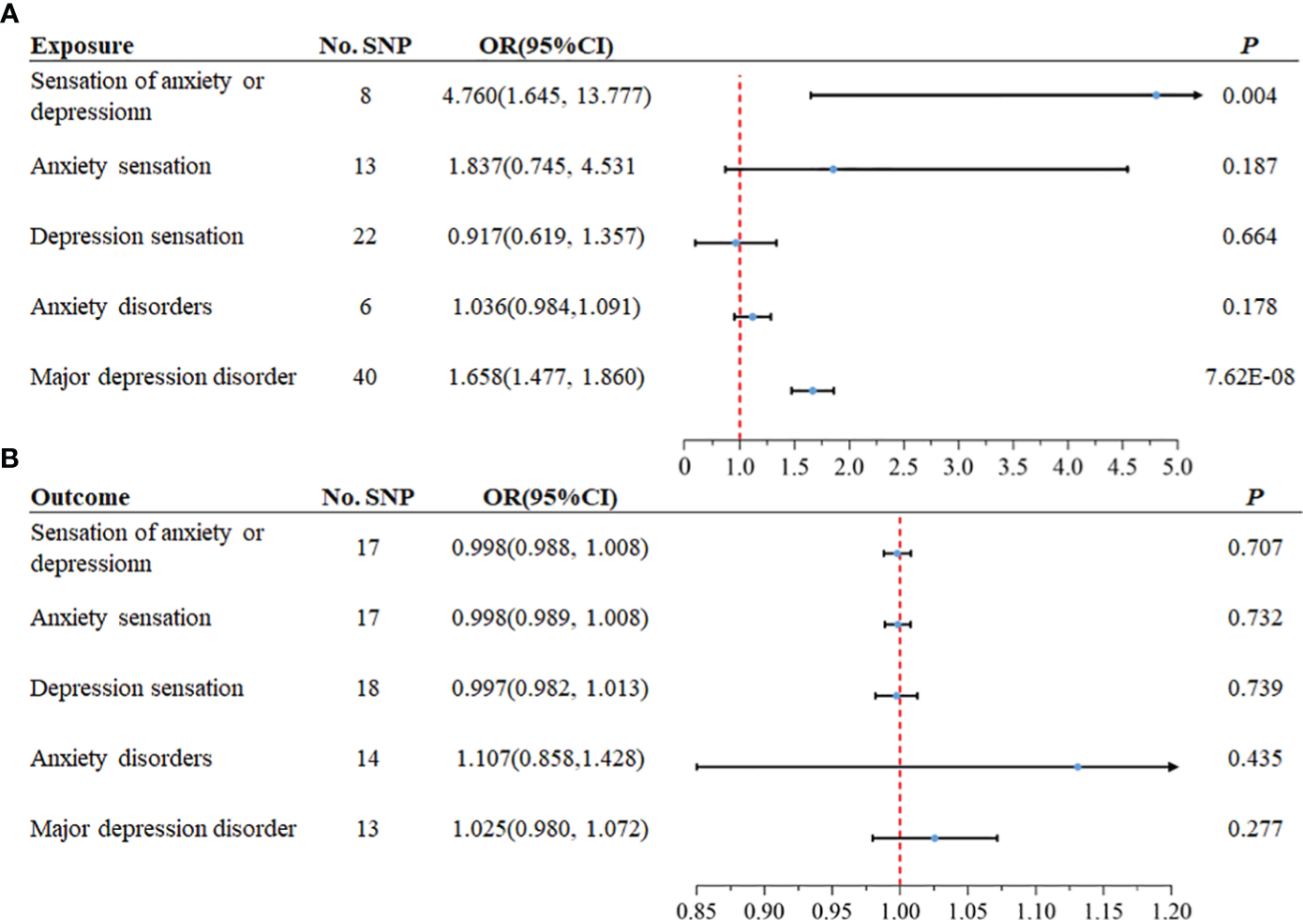
The MR analysis results between mental disorders and asthma. In both forward and reverse Mendelian Randomization (MR), Inverse Variance Weighted (IVW) method is primarily employed. **(A)** The causal effect of anxiety and depression on asthma (forward MR), **(B)** The causal effect of asthma on anxiety and depression (reverse MR).

**Figure 4:**
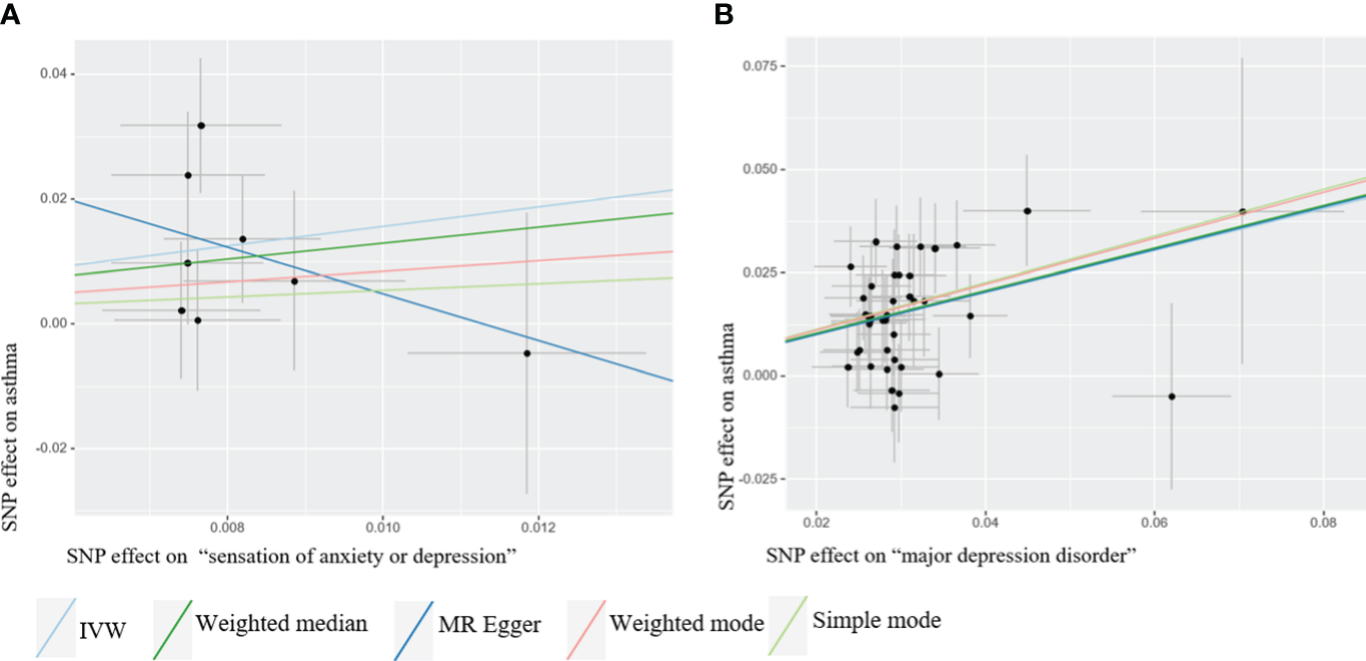
Scatter plots of Mendelian Randomization (MR) where exposure is causally related to outcome. The MR analysis employed five methods, namely inverse variance weighted (IVW), weighted median, MR-Egger regression, simple mode, and weighted mode. **(A)** “Sensation of anxiety or depression” as exposure **(B)** Major depression disorder (MDD) as exposure.

We identified a causal relationship between sensation of anxiety or depression, MDD, and asthma. We observed that genetically predicted sensation of anxiety or depression was positively associated with the risk of asthma (OR =4.760, 95%CI: 1.645–13.777, *P* =0.004), and MDD was also positively associated with the risk of asthma (OR =1.658, 95%CI: 1.477–1.860, *P* =7.62×10–^18^). In contrast, we discovered no evidence that genetically predicted anxiety sensation was associated with the risk of asthma (OR = 1.837, 95% CI: 0.745–4.531, *P* = 0.187), genetically predicted depression sensation was associated with the risk of asthma (OR = 0.917, 95% CI: 0.619–1.357, *P* = 0.664), or genetically predicted anxiety disorders were associated with the risk of asthma (OR = 1.036, 95% CI: 0.984–1.091, *P* = 0.178), as displayed in **Figure 3**. **Figure 4** presents the results of the scatter plots for outcomes causally related to asthma. The heterogeneity analysis yielded results indicating uniformity across the IVs, as evidenced in **Supplementary Table S2**. Furthermore, the robustness of the MR analysis was confirmed through sensitivity analysis employing the leave-one-out method, as shown in **Supplementary Figure S1**.

### 3.3 The causal effect of asthma on anxiety and depression (reverse MR)

We extracted 19 SNPs from the exposure data. Finally, 17, 17, 18, 14, and 13 SNPs were employed, each demonstrating an F statistic >10, as depicted in **Figure 2**. Following the removal of an outlier from the variable associated with MDD as the outcome, no evidence of pleiotropy was observed in the subsequent pleiotropy test. Consequently, the MR analysis proceeded using the IVW method as the primary approach.

The analysis suggested there was no association between genetically predicted asthma and the risk of developing anxiety disorders (OR =1.107, 95%CI: 0.858–1.428, *P* =0.435). Similarly, our analysis found no significant association between elevated genetically predicted asthma and the increased risk of other mental states, as illustrated in **Figure 3**. The heterogeneity analysis revealed consistent results among the instrumental variables (**Supplementary Table S2**). Moreover, the MR analysis’s robustness was substantiated by a sensitivity analysis using the leave-one-out method (**Supplementary Figure S2**).

### 3.4 MVMR analysis of MDD’s causal impact on asthma

Multivariate MR, adjusted for BMI and alcohol consumption, reinforced the independent causal impact of MDD on asthma risk, albeit with a marginally reduced effect size (OR =1.460, 95% CI: 1.285 -1.660, *P*=7.05×10–^9^). Given the heterogeneity in IVW results, we applied the MR-Egger and weighted median methods. These methods corroborated the initial findings, showing enhanced results, as outlined in **Table 2**.

**Table 2:** The result of multivariate MR (MVMR) analysis for MDD's causal impact on asthma.

Exposure	Outcome	Method	No. SNP	OR (95%CI)	*P*
MDD, BMI and alcohol consumption	Asthma	IVW	481	1.460 (1.285, 1.660)	7.05E-09
		MR-Egger	481	1.457 (1.281, 1.656)	9.34E-09
		weighted median	481	1.608 (1.381, 1.872)	9.57E-10

MDD, major depression disorder; BMI, body mass index; IVW, inverse variance weighted.

## 4 Discussion

Using comprehensive GWAS data, we systematically examined the bidirectional link between common mental disorders and asthma. Our preliminary results suggested a shared genetic foundation among sensation of anxiety or depression, anxiety disorders, MDD and asthma, including those with a potential correlation. Additionally, our two-sample MR studies indicated a unidirectional causal effect of genetic predisposition to MDD on asthma, which was further affirmed by multivariate MR adjustments. Concurrently, our research also indicated that anxiety disorders are not causal risk factors for asthma, and likewise, the reverse is not true.

Anwar et al. (35), utilizing a phenome-wide association study (PheWAS) followed by MR, and Zhu et al. (18), via generalized summary data MR, both uncovered evidence for the causal effects of MDD on the risk of asthma. Our MR analysis not only is in line with the existing evidence regarding MDD but also distinctively emphasizes its substantial causal impact on asthma, enhancing the previous MR study (34) in three major aspects. Initially, we classified depression into two severity-based categories leading to more precise results. It revealed that specifically the clinical state of depression, which reached the diagnosis of MDD, rather than mere feelings, is causally linked to asthma. Second, the prior MR study’s GWAS data for depression and asthma had a 6.7% sample overlap, posing a risk of type I error. Our study used distinct, updated GWAS datasets, enhancing the validity of our results. Third, our study expanded the MR analysis to include a comprehensive framework with sensitivity analyses for directional pleiotropy and a multivariate MR approach, providing an independent causal assessment. The evidence supporting a causal effect of MDD on asthma is increasingly compelling.

Multiple mechanisms could elucidate the link between MDD and asthma. Depression may cause vagally mediated bronchoconstriction or increased interoceptive sensitivity, accelerating asthma development (36, 37). Chen proposed that depression might intensify airway inflammation in response to environmental triggers (38). Key biological pathways involved are the hypothalamic-pituitary-adrenal (HPA) axis, the sympathetic-adrenal-medullary (SAM) axis, and the sympathetic (SNS) and parasympathetic (PNS) arms of the autonomic nervous system. Depression can activate the SNS, triggering epinephrine release and stimulating noradrenergic fibers in lymphoid tissues. Released catecholamines binding to lymphocytes may enhance humoral responses (39). Concurrently, adrenergic receptors may trigger mast cells to release histamines and activate eosinophils in the airways. Iwata et al. (40) suggested the NOD-like receptor family pyrin domain-containing 3 (NLRP3) inflammasome in inflammatory signal transduction as a potential link between depression and asthma. Secondly, depression might elevate asthma risk through lifestyle factors like smoking and poor health management, and depression may also impact lung function directly, possibly through hyperventilation mechanisms (41, 42). Thirdly, Zhu et al. (34) identified a genetic link between depression and asthma, with an overlap in the POLI gene, suggesting a role for DNA polymerase iota (η) enzyme in this relationship.

A previous study (11) observed a genetic correlation between asthma and depression using LDSC, yet it did not find the connection between asthma and anxiety. On the other hand, Zhu et al. (34) encountered limitations due to a lack of significant SNPs in their anxiety GWAS for adequate MR analysis. Similarly, Ashley et al. (43) applied MR analysis but could not confirm a causal link from asthma to MDD or anxiety. Utilizing updated datasets, our research successfully carried out genetic analysis and identified a potential genetic correlation between asthma and anxiety disorders through LDSC. Furthermore, our study suggests that there is no causal effect of asthma on anxiety disorders, as evident from MR analysis, contrasting with earlier observational studies. Potential unmeasured confounders in earlier studies might explain this discrepancy. Thought there are several possible mechanisms for asthma’s effect on anxiety disorders include: 1) Activation of the mPFC-amygdala circuit by neuroimmune responses (44), and 2) Enhancement of microglia and astrocytes by Th2 inflammatory factors, increasing neural oscillations (45), however, additional research is warranted to solidify this causal association. Additionally, while focusing on shared genetic factors, our study was not able to account for environmental or other non-genetic factors, such as asthma awareness potentially contributing to increased anxiety. Further investigation is required to elucidate the causal dynamics. Moreover, depression and anxiety have overlapping regulatory pathways in asthma pathogenesis, like oxidative stress and inflammation (46), which may influence their causal interrelations.

It’s important to acknowledge several limitations in our study. Firstly, asthma, anxiety, and depression are heterogeneous, with correlations influenced by various factors like gender and age; for example, females with severe asthma often tend to have higher rates of anxiety and depression than males (47). Additionally, the bidirectional relationship between depression, anxiety, and asthma not only exists in adults but also extends to children with asthma (48). Our understanding is still limited, which necessitates future studies with larger sample sizes to conduct more detailed subgroup analysis of these diseases. Second, using European population data to reduce stratification bias may limit generalizability to other populations. Conclusively verifying these relationships demands further analyses, including gene function studies and longitudinal research.

## 5 Conclusion

In conclusion, large-scale genetic studies reveal a significant causal effect of MDD on asthma and indicate that anxiety disorders are not causal risk factors for asthma, nor is the reverse true. These findings highlight the importance of MDD prevention in reducing asthma incidence. This research contributes to advancing our understanding of disease prevention and the management of complications.

## Data availability statement

Publicly available datasets were analyzed in this study. The accession websites and GWAS ID for the datasets can be found in **Supplementary Material Table S1**. Further inquiries can be directed to the corresponding author.

## Author contributions

ZY: Writing – original draft, Writing – review & editing, Software. JC: Funding acquisition, Investigation, Visualization, Writing – review & editing. LG: Project administration, Writing – review & editing. HZ: Visualization, Writing – review & editing. YD: Visualization, Writing – review & editing. GR: Visualization, Writing – review & editing. YM: Visualization, Writing – review & editing. RB: Visualization, Writing – review & editing. XM: Project administration, Writing – review & editing.

## References

[1] GBD 2019 Mental Disorders Collaborators. Global, regional, and national burden of 12 mental disorders in 204 countries and territories, 1990–2019: A systematic analysis for the global burden of disease study 2019. Lancet Psychiatry. (2022) 9:137–50. doi: 10.1016/S2215-0366(21)00395-3, PMID: 35026139 PMC8776563

[2] Groen RN Ryan O Wigman JTW Riese H Penninx BWJH Giltay EJ. Comorbidity between depression and anxiety: Assessing the role of bridge mental states in dynamic psychological networks. BMC Med. (2020) 18:308. doi: 10.1186/s12916-020-01738-z, PMID: 32988400 PMC7523307

[3] Szuhany KL Simon NM. Anxiety disorders: A review. Jama. (2022) 328:2431–45. doi: 10.1001/jama.2022.22744, PMID: 36573969

[4] Kessler RC Petukhova M Sampson NA Zaslavsky AM Wittchen HU. Twelve-month and lifetime prevalence and lifetime morbid risk of anxiety and mood disorders in the United States. Int J Meth Psych Res. (2012) 21:169–84. doi: 10.1002/mpr.1359, PMID: 22865617 PMC4005415

[5] Cruz-Pereira JS Rea K Nolan YM O’Leary OF Dinan TG Cryan JF. Depression’s unholy trinity: Dysregulated stress, immunity, and the microbiome. Annu Rev Psychol. (2020) 71:49–78. doi: 10.1146/annurev-psych-122216-011613, PMID: 31567042

[6] Stern J Pier J Litonjua AA. Asthma epidemiology and risk factors. Semin Immunopathol. (2020) 42:5–15. doi: 10.1007/s00281-020-00785-1, PMID: 32020334

[7] GBD 2015 Chronic Respiratory Disease Collaborators. Global, regional, and national deaths, prevalence, disability-adjusted life years, and years lived with disability for chronic obstructive pulmonary disease and asthma, 1990–2015: A systematic analysis for the global burden of disease study 2015. Lancet Respir Med. (2017) 5:691–706. doi: 10.1016/S2213-2600(17)30293-X, PMID: 28822787 PMC5573769

[8] Moussavi S Chatterji S Verdes E Tandon A Patel V Ustun B. Depression, chronic diseases, and decrements in health: Results from the world health surveys. Lancet (London England). (2007) 370:851–8. doi: 10.1016/S0140-6736(07)61415-9, PMID: 17826170

[9] Skou ST Mair FS Fortin M Guthrie B Nunes BP Miranda JJ. Multimorbidity. Nat Rev Dis Primers. (2022) 8:48. doi: 10.1038/s41572-022-00376-4, PMID: 35835758 PMC7613517

[10] Scott KM Von Korff M Ormel J Zhang MY Bruffaerts R Alonso J. Mental disorders among adults with asthma: Results from the world mental health survey. Gen Hosp Psychiatry. (2007) 29:123–33. doi: 10.1016/j.genhosppsych.2006.12.006, PMID: 17336661 PMC1913936

[11] Lehto K Pedersen NL Almqvist C Lu Y Brew BK. Asthma and affective traits in adults: A genetically informative study. Eur Respir J. (2019) 53:1802142. doi: 10.1183/13993003.02142-2018, PMID: 30956207

[12] Ye G Baldwin DS Hou R. Anxiety in asthma: A systematic review and meta-analysis. Psychol Med. (2021) 51:11–20. doi: 10.1017/S0033291720005097, PMID: 33431086

[13] Swanson SA Tiemeier H Ikram MA Hernán MA. Nature as a trialist?: deconstructing the analogy between Mendelian randomization and randomized trials. Epidemiology. (2017) 28:653–9. doi: 10.1097/EDE.0000000000000699, PMID: 28590373 PMC5552969

[14] Richmond RC Davey Smith G. Mendelian randomization: concepts and scope. Cold Spring Harb Perspect Med. (2022) 12:a040501. doi: 10.1101/cshperspect.a040501, PMID: 34426474 PMC8725623

[15] Hu S Xing H Wang X Zhang N Xu Q. Causal relationships between total physical activity and ankylosing spondylitis: A mendelian randomization study. Front Immunol. (2022) 13:887326. doi: 10.3389/fimmu.2022.887326, PMID: 35865535 PMC9294357

[16] Burgess S Davies NM Thompson SG. Bias due to participant overlap in two-sample Mendelian randomization. Genet Epidemiol. (2016) 40:597–608. doi: 10.1002/gepi.21998, PMID: 27625185 PMC5082560

[17] Kurki MI Karjalainen J Palta P Sipilä TP Kristiansson K Donner KM. FinnGen provides genetic insights from a well-phenotyped isolated population. Nature. (2023) 613:508–18. doi: 10.1038/s41586-022-05473-8, PMID: 36653562 PMC9849126

[18] Burgess S Butterworth A Thompson SG. Mendelian randomization analysis with multiple genetic variants using summarized data. Genet Epidemiol. (2013) 37:658–65. doi: 10.1002/gepi.21758, PMID: 24114802 PMC4377079

[19] Zhou Q Jin X Li H Wang Q Tao M Wang J. Cholesterol and low-density lipoprotein as a cause of psoriasis: results from bidirectional mendelian randomization. J Eur Acad Dermatol Venereol. (2024) 38:710–8. doi: 10.1111/jdv.19670, PMID: 38031463

[20] Bulik-Sullivan B Finucane HK Anttila V Gusev A Day FR Loh PR. An atlas of genetic correlations across human diseases and traits. Nat Genet. (2015) 47:1236–41. doi: 10.1038/ng.3406, PMID: 26414676 PMC4797329

[21] Li GHY Cheung CL Chung AKK Cheung BMY Wong ICK Fok MLY. Evaluation of bi-directional causal association between depression and cardiovascular diseases: a Mendelian randomization study. Psychol Med. (2022) 52:1765–76. doi: 10.1017/S0033291720003566, PMID: 33032663

[22] Ellingjord-Dale M Papadimitriou N Katsoulis M Yee C Dimou N Gill D. Coffee consumption and risk of breast cancer: A Mendelian randomization study. PloS One. (2021) 16:e0236904. doi: 10.1371/journal.pone.0236904, PMID: 33465101 PMC7815134

[23] Huang S Tian F Yang X Fang S Fan Y Bao J. Physical activity and systemic lupus erythematosus among European populations: A two-sample Mendelian randomization study. Front Genet. (2022) 12:784922. doi: 10.3389/fgene.2021.784922, PMID: 35211151 PMC8861300

[24] Sang N Gao RC Zhang MY Wu ZZ Wu ZG Wu GC. Causal relationship between sleep traits and risk of systemic Lupus erythematosus: A two-sample Mendelian randomization study. Front Immunol. (2022) 13:918749. doi: 10.3389/fimmu.2022.918749, PMID: 35784289 PMC9248809

[25] Bowden J Spiller W Del Greco MF Sheehan N Thompson J Minelli C. Improving the visualization, interpretation and analysis of two-sample summary data Mendelian randomization via the Radial plot and Radial regression. Int J Epidemiol. (2018) 47:2100–0. doi: 10.1093/ije/dyy265, PMID: 30423109 PMC6280936

[26] Burgess S Thompson SG. Multivariable Mendelian randomization: the use of pleiotropic genetic variants to estimate causal effects. Am J Epidemiol. (2015) 181:251–60. doi: 10.1093/aje/kwu283, PMID: 25632051 PMC4325677

[27] Mikkelsen H Landt EM Benn M Nordestgaard BG Dahl M. Causal risk factors for asthma in mendelian randomization studies: A systematic review and meta-analysis. Clin Trans Allergy. (2022) 12:e12207. doi: 10.1002/clt2.12207, PMID: 36434743 PMC9640961

[28] 23andMe Research Team HUNT All-In Psychiatry Liu M Jiang Y Wedow R Li Y. Association studies of up to 1.2 million individuals yield new insights into the genetic etiology of tobacco and alcohol use. Nat Genet. (2019) 51:237–44. doi: 10.1038/s41588-018-0307-5, PMID: 30643251 PMC6358542

[29] Pulit SL Stoneman C Morris AP Wood AR Glastonbury CA Tyrrell J. Meta-analysis of genome-wide association studies for body fat distribution in 694 649 individuals of European ancestry. Hum Mol Genet. (2019) 28:166–74. doi: 10.1093/hmg/ddy327, PMID: 30239722 PMC6298238

[30] Rees JMB Wood AM Burgess S. Extending the MR-Egger method for multivariable Mendelian randomization to correct for both measured and unmeasured pleiotropy. Stat Med. (2017) 36:4705–18. doi: 10.1002/sim.7492, PMID: 28960498 PMC5725762

[31] Yavorska OO Burgess S. MendelianRandomization: An R package for performing mendelian randomization analyses using summarized data. Int J Epidemiol. (2017) 46:1734–9. doi: 10.1093/ije/dyx034, PMID: 28398548 PMC5510723

[32] Patel A Ye T Xue H Lin Z Xu S Woolf B. MendelianRandomization v0.9.0: Updates to an R package for performing mendelian randomization analyses using summarized data. Wellcome Open Res. (2023) 8:449. doi: 10.12688/wellcomeopenres, PMID: 37915953 PMC10616660

[33] Armstrong RA. When to use the bonferroni correction. Ophthalmic Physiol Opt: J Br Coll Ophthalmic Opt (Optom). (2014) 34:502–8. doi: 10.1111/opo.12131, PMID: 24697967

[34] Zhu Z Zhu X Liu CL Shi H Shen S Yang Y. Shared genetics of asthma and mental health disorders: A large-scale genome-wide cross-trait analysis. Eur Respir J. (2019) 54:1901507. doi: 10.1183/13993003.01507-2019, PMID: 31619474

[35] Mulugeta A Zhou A King C Hyppönen E. Association between major depressive disorder and multiple disease outcomes: a phenome-wide Mendelian randomisation study in the UK Biobank. Mol Psychiatry. (2020) 25:1469–76. doi: 10.1038/s41380-019-0486-1, PMID: 31427754

[36] Wright R. Stress and atopic disorders. J Allergy Clin Immunol. (2005) 116:1301–6. doi: 10.1016/j.jaci.2005.09.050, PMID: 16337463

[37] Wright RJ Rodriguez M Cohen S. Review of psychosocial stress and asthma: an integrated biopsychosocial approach. Thorax. (1998) 53:1066–74. doi: 10.1136/thx.53.12.1066, PMID: 10195081 PMC1745142

[38] Chen E Miller GE. Stress and inflammation in exacerbations of asthma. Brain Behav Immun. (2007) 21:993–9. doi: 10.1016/j.bbi.2007.03.009, PMID: 17493786 PMC2077080

[39] Marshall GD Agarwal SK. Stress, immune regulation, and immunity: applications for asthma. Allergy Asthma Proc. (2000) 21:241–6. doi: 10.2500/108854100778248917, PMID: 10951892

[40] Iwata M Ota KT Duman RS. The inflammasome: Pathways linking psychological stress, depression, and systemic illnesses. Brain Behavior Immunity. (2013) 31:105–14. doi: 10.1016/j.bbi.2012.12.008, PMID: 23261775 PMC4426992

[41] Chan A Yii A Tay CK Lapperre T Tan LL Yeoh F. The impact of anxiety and depression on asthma-related health outcomes: a prospective study. Allergy Immunol. (2015), PA5097. doi: 10.1183/13993003.congress-2015.PA5097, PMID: 40074277

[42] Cleveland Clinic. Smoking & asthma: effects, prevention. Available online at: https://my.clevelandclinic.org/health/articles/4584-smoking–asthma.

[43] Budu-Aggrey A Joyce S Davies NM Paternoster L Munafò MR Brown SJ. Investigating the causal relationship between allergic disease and mental health. Clin Exp Allergy: J Br Soc Allergy Clin Immunol. (2021) 51:1449–58. doi: 10.1111/cea.14010, PMID: 34611950

[44] Liu WZ Zhang WH Zheng ZH Zou JX Liu XX Huang SH. Identification of a prefrontal cortex-to-amygdala pathway for chronic stress-induced anxiety. Nat Commun. (2020) 11:2221. doi: 10.1038/s41467-020-15920-7, PMID: 32376858 PMC7203160

[45] Gholami-Mahtaj L Mooziri M Dehdar K Abdolsamadi M Salimi M Raoufy MR. ACC-BLA functional connectivity disruption in allergic inflammation is associated with anxiety. Sci Rep. (2022) 12:2731. doi: 10.1038/s41598-022-06748-w, PMID: 35177766 PMC8854589

[46] Jiang M Qin P Yang X. Comorbidity between depression and asthma via immune-inflammatory pathways: A meta-analysis. J Affect Disord. (2014) :166:22–9. doi: 10.1016/j.jad.2014.04.027, PMID: 25012406

[47] Anastasia P Eleni T Eleftheria M Xenia N Eygenia P Kyriakos S. Depression levels influence the rate of asthma exacerbations in females. J Personalized Med. (2021) 11:586. doi: 10.3390/jpm11060586, PMID: 34205619 PMC8235599

[48] Brew BK Osvald EC Gong T Hedman AM Holmberg K Larsson H. Paediatric asthma and non-allergic comorbidities: A review of current risk and proposed mechanisms. Clin Exp Allergy: J Br Soc Allergy Clin Immunol. (2022) 52:1035–47. doi: 10.1111/cea.14207, PMID: 35861116 PMC9541883

