# Supplementary material for: Genetic analyses of the bidirectional associations between common mental disorders and asthma
Source: Front Psychiatry. 2024 Jun 6;15:1372842. doi: 10.3389/fpsyt.2024.1372842 (PMC11187307; doi:10.3389/fpsyt.2024.1372842)
Supplement: Supplementary file 1 [file DataSheet1.pdf]

• *Supplementary Material*

Supplementary Figures and Tables

1 Supplementary Figures

Figure S1 Results of leave-one-out method.

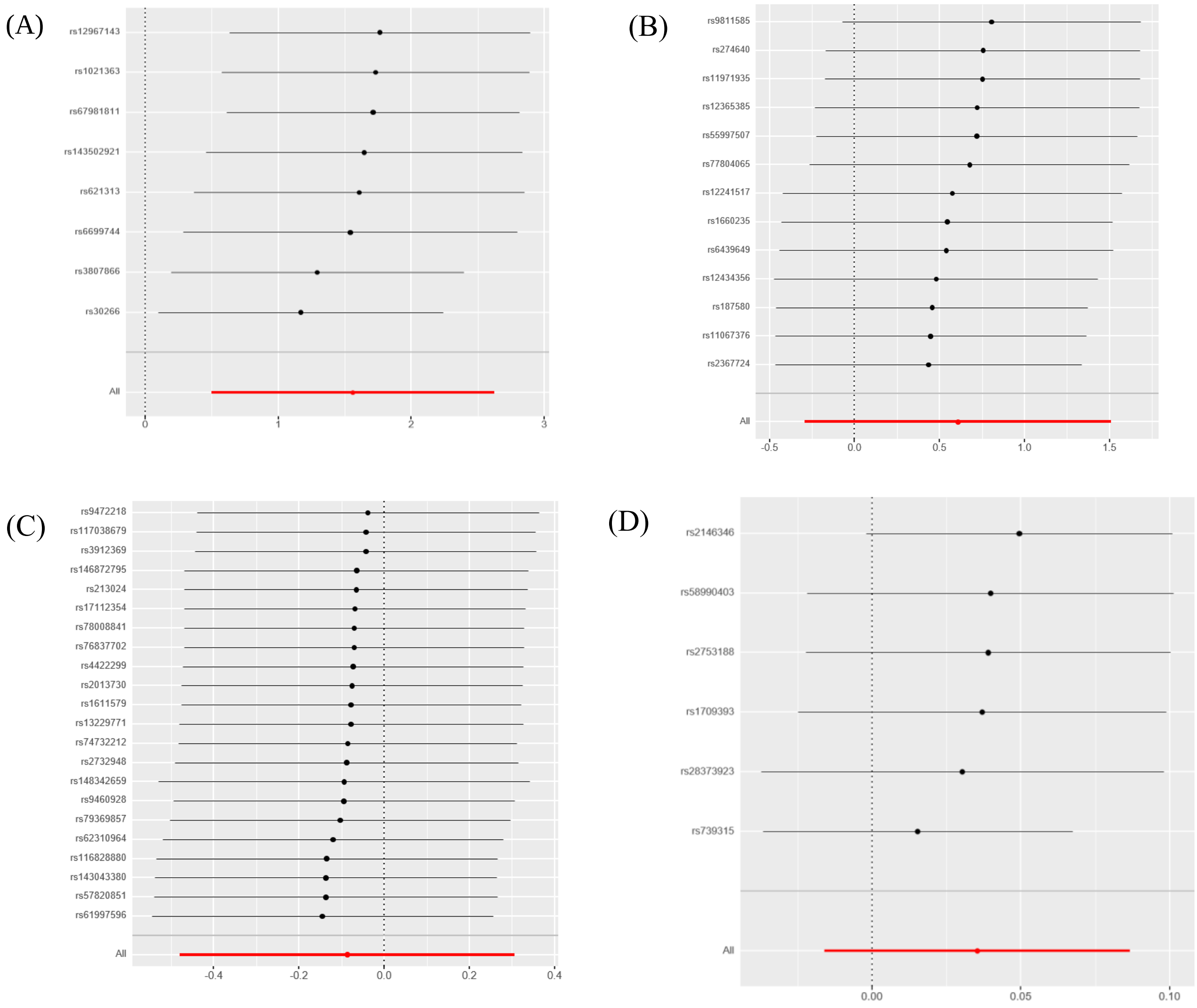

(E)

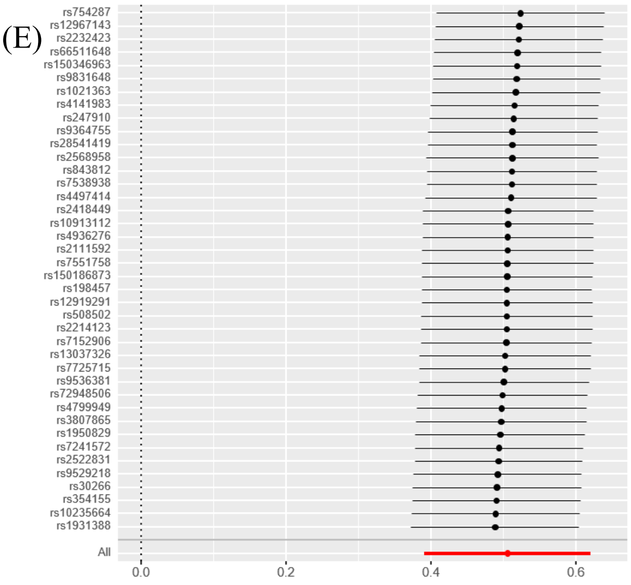
rs754287
rs12967143
rs2232423
rs66511648
rs150346963
rs9831648
rs1021363
rs4141983
rs247910
rs9364755
rs28541419
rs2568958
rs843812
rs7538938
rs4497414
rs2418449
rs10913112
rs4936276
rs2111592
rs7551758
rs150186873
rs198457
rs12919291
rs508502
rs2214123
rs7152906
rs13037326
rs7725715
rs9536381
rs72948506
rs4799949
rs3807865
rs1950829
rs7241572
rs2522831
rs9529218
rs30266
rs354155
rs10235664
rs1931388
All
0.0
0.2
0.4
0.6

(F)

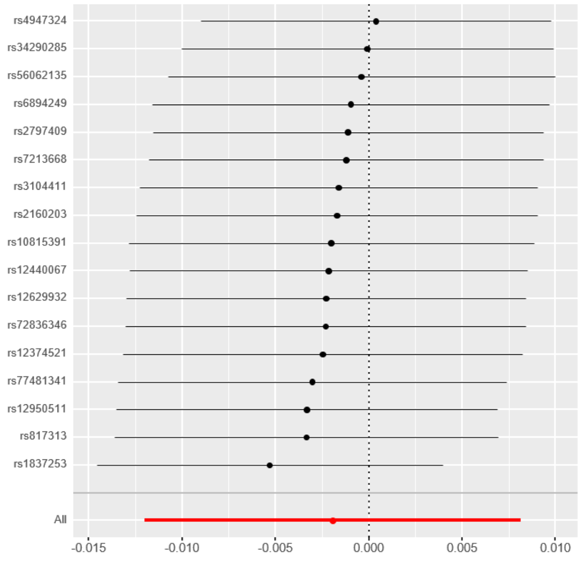
rs4947324
rs34290285
rs56062135
rs6894249
rs2797409
rs7213668
rs3104411
rs2160203
rs10815391
rs12440067
rs12629932
rs72836346
rs12374521
rs77481341
rs12950511
rs817313
rs1837253
All
-0.015
-0.010
-0.005
0.000
0.005
0.010

(G)

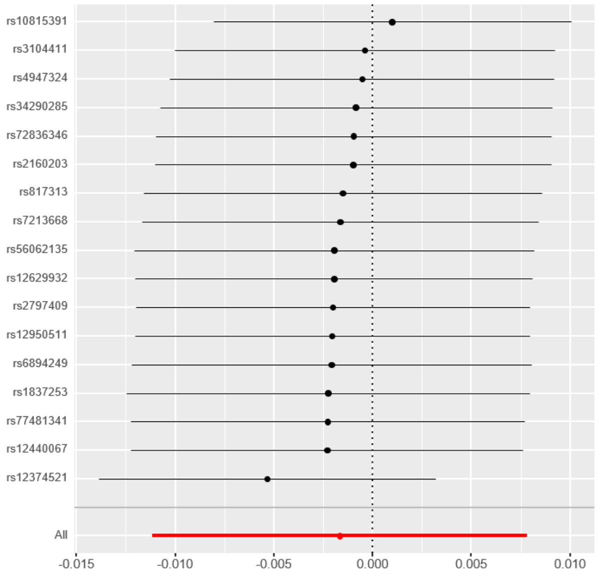
rs10815391
rs3104411
rs4947324
rs34290285
rs72836346
rs2160203
rs817313
rs7213668
rs56062135
rs12629932
rs2797409
rs12950511
rs6894249
rs1837253
rs77481341
rs12440067
rs12374521
All
-0.015
-0.010
-0.005
0.000
0.005
0.010

(H)

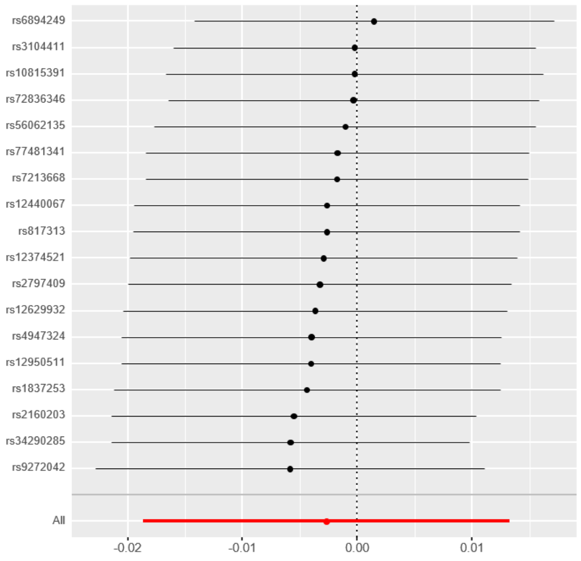
rs6894249
rs3104411
rs10815391
rs72836346
rs56062135
rs77481341
rs7213668
rs12440067
rs817313
rs12374521
rs2797409
rs12629932
rs4947324
rs12950511
rs1837253
rs2160203
rs34290285
rs9272042
All
-0.02
-0.01
0.00
0.01

(I)

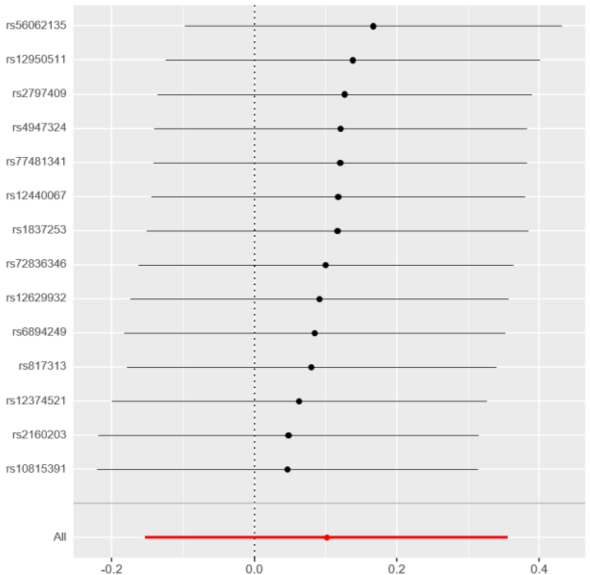
rs56062135
rs12950511
rs2797409
rs4947324
rs77481341
rs12440067
rs1837253
rs72836346
rs12629932
rs6894249
rs817313
rs12374521
rs2160203
rs10815391
All
-0.2
0.0
0.2
0.4

(J)

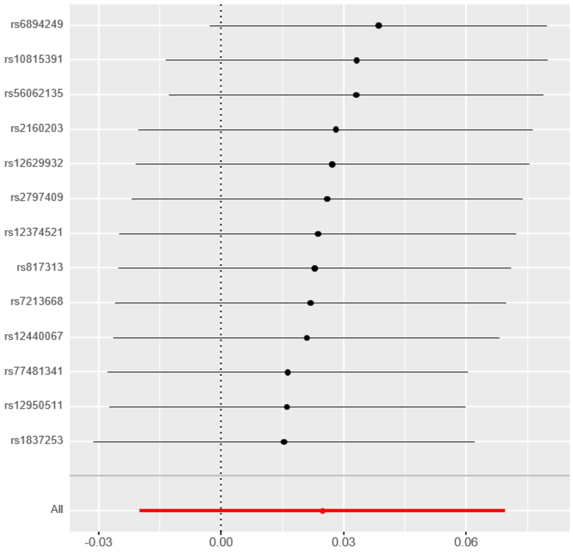
rs6894249
rs10815391
rs56062135
rs2160203
rs12629932
rs2797409
rs12374521
rs817313
rs7213668
rs12440067
rs77481341
rs12950511
rs1837253
All
-0.03
0.00
0.03
0.06

(A) Asthma as outcome and “sensation of anxiety or depression” as exposure,;
(B) Asthma as outcome and “anxiety sensation” as exposure;
(C) Asthma as outcome and “depression sensation” as exposure;
(D) Asthma as outcome and “anxiety disorders” as exposure;
(E) Asthma as outcome and “major depression disorder” as exposure;
(F) Asthma as exposure and “sensation of anxiety or depression” as outcome;
(G) Asthma as exposure and “anxiety sensation” as outcome;
(H) Asthma as exposure and “depression sensation” as outcome;
(I) Asthma as exposure and “anxiety disorders” as outcome;
(J) Asthma as exposure and “major depression disorder” as outcome.

2 Supplementary Tables

Table S1 Data source for common mental disorders and asthma

Phenotype	Consortium/ GWAS ID/ PMID	Original trait name	Population	Sample size	SNPs
Sensation of anxiety or depression	ukb-b-6991	Seen doctor (GP) for nerves, anxiety, tension or depression	European	459560	9851867
Anxiety sensation	ukb-b-6519	Worrier/ anxious feelings	European	450765	9851867
Depression sensation	ukb-d-20446	Ever had prolonged feelings of sadness or depression	European	117763	13571578
Anxiety disorders	26754954	/*	European	17310	6330995
Major depression disorder	30718901	/*	European	807553	8098588
Asthma	FinnGen biobank (R10)	/*	European	251919	19341428
Body mass index	30239722	/*	European	806843	/
Alcohol consumption	30643251	/*	European	941280	/

*: The names of these traits have not been recategorized and remain as listed in the "phenotype" section. Asthma diagnoses in the FinnGen dataset are based on ICD-10 code J45.9. The genome-wide meta-analysis for major depression disorder (MDD) is based on the three largest studies. These encompass broad depression, probable MDD identified through self-reported symptoms with associated impairment, and MDD determined from hospital

admission records, et al. The genome-wide meta-analysis for anxiety disorders includes phenotypes such as generalized anxiety disorder (GAD), panic disorder (PD), social phobia, agoraphobia, and specific phobias.

Table S2 Heterogeneity and pleiotropy tests of the two-sample bidirectional Mendelian randomization (MR) analysis

Exporsure	Outcome	Heterogeneity test	*P*	Pleiotropy test	*P*
Sensation of anxiety or depression	Asthma	MR Egger	0.349	MR Egger	0.338*
		IVW	0.340	MR-PRESSO	/
Anxiety sensation		MR Egger	0.177	MR Egger	0.342
		IVW	0.170	MR-PRESSO	0.196
Depression sensation		MR Egger	0.902	MR Egger	0.637
		IVW	0.921	MR-PRESSO	0.930
Anxiety disorders		MR Egger	0.231	MR Egger	0.792
		IVW	0.335	MR-PRESSO	0.393
Major depression disorder		MR Egger	0.394	MR Egger	0.975*
		IVW	0.439	MR-PRESSO	/
Asthma	Sensation of anxiety or depression	MR Egger	0.036	MR Egger	0.913
		IVW	0.051	MR-PRESSO	0.058
	Anxiety sensation	MR Egger	0.239	MR Egger	0.170
		IVW	0.178	MR-PRESSO	0.175
	Depression sensation	MR Egger	0.284	MR Egger	0.935
		IVW	0.345	MR-PRESSO	0.346
	Anxiety disorders	MR Egger	0.550	MR Egger	0.949
		IVW	0.631	MR-PRESSO	0.631
		MR Egger	0.163	MR Egger	0.942*

	Major depression disorder	IVW	0.217	MR-PRESSO	/

*: In these analyses, outliers were initially identified by MR-PRESSO in the preliminary analysis. Subsequently, horizontal pleiotropy was reassessed using MR-Egger test in the formal analysis, following the removal of these outliers.

3 Supplementary Data

DataTable S1 The SNPs used as genetic instruments in the Mendelian randomization (MR) analyses for the outcome of asthma, and the exposures are "sensation of anxiety or depression", "anxiety sensation", "depression sensation", "anxiety disorders", and "major depression disorder", respectively.

(K) Asthma as outcome and "sensation of anxiety or depression" as exposure.

SNPs	effect_allele	other_allele	β. exposure	β.outcome	eaf. exposure	eaf. outcome	se.exposure	se.outcome	*P*.exposure	*P*.outcome	F
rs1021363	G	A	-0.00740736	-0.00217394	0.643587	0.725952	0.00102059	0.010957	3.90E-13	0.843	11.568
rs12967143	C	G	-0.00761209	-0.000520463	0.699435	0.746183	0.00107088	0.0112327	1.20E-12	0.963	11.196
rs143502921	C	G	0.00885943	0.00687477	0.179546	0.139795	0.00142321	0.0143797	4.80E-10	0.633	10.627
rs30266	A	G	0.00765851	0.0317453	0.32822	0.289686	0.00104038	0.0107575	1.80E-13	0.003	11.887
rs3807866	A	G	0.0074939	0.0238351	0.410746	0.363959	0.000990903	0.01014	3.90E-14	0.019	12.493
rs621313	G	A	0.00748553	0.00967759	0.49123	0.550722	0.000979968	0.0098315	2.20E-14	0.325	12.872
rs6699744	T	A	0.00819286	0.0135801	0.615966	0.645396	0.00100769	0.0102235	4.30E-16	0.184	14.594
rs67981811	G	C	-0.011852	0.00472773	0.11416	0.0503637	0.00153202	0.0225638	1.00E-14	0.834	13.057

(L) Asthma as outcome and "anxiety sensation" as exposure.

SNP	effect_allele	other_allele	β. exposure	β.outcome	eaf. exposure	eaf. outcome	se.exposure	se.outcome	*P*.exposure	*P*.outcome	F
rs11067376	G	A	0.00732746	0.0192379	0.359672	0.307714	0.00106892	0.0105794	7.1007E-12	0.069	11.14821
rs11971935	G	T	-0.0071302	0.0087974	0.407609	0.322877	0.00104471	0.0104387	8.8004E-12	0.399	11.06747
rs12241517	G	A	-0.0087792	-0.00778287	0.328838	0.324748	0.00109126	0.0104285	8.6E-16	0.455	15.33615
rs12365385	C	T	-0.0070752	0.00491553	0.365973	0.446424	0.00106653	0.0098481	3.2999E-11	0.618	10.47192
rs12434356	A	C	0.0073884	0.0143614	0.520632	0.49408	0.00102523	0.0097715	5.7003E-13	0.142	12.28261
rs1660235	G	C	0.00729017	0.00976505	0.386462	0.379057	0.00106016	0.0100964	6.0996E-12	0.333	11.3609
rs187580	G	T	-0.0078221	-0.0204664	0.237953	0.233679	0.00120706	0.0115529	9.2003E-11	0.076	10.00245
rs2367724	T	C	0.00716112	0.0200795	0.67278	0.672922	0.00109177	0.0103854	5.4001E-11	0.053	10.17801
rs274640	A	G	-0.0068616	0.00866885	0.414121	0.396484	0.0010403	0.0099978	4.1995E-11	0.386	10.29862
rs55997507	C	G	0.00694102	-0.00622567	0.609043	0.678143	0.00105979	0.0104306	5.7996E-11	0.551	10.34217
rs6439649	T	G	0.00781929	0.00975908	0.601407	0.461959	0.00104552	0.0098123	7.5007E-14	0.320	13.21368
rs77804065	T	C	0.00812236	-0.008316	0.225027	0.10546	0.00123652	0.0159807	5.1004E-11	0.603	10.37227
rs9811585	G	T	0.00733612	-0.0140295	0.380805	0.295951	0.00105656	0.010725	3.8001E-12	0.191	11.44069

(M) Asthma as outcome and “depression sensation” as exposure.

SNP	Effect _allele	Other _allele	β. exposure	β.outcome	eaf. exposure	eaf. outcome	se.exposure	se.outcome	*P*.exposure	*P*.outcome	F*
rs116828880	T	C	0.021171	0.023936	0.06253	0.0549763	0.00425362	0.0216837	6.4607E-07	0.270	24.772
rs117038679	T	C	-0.032763	0.0609565	0.022245	0.0136669	0.00692128	0.0421101	2.2076E-06	0.148	22.408
rs13229771	C	T	0.015852	-0.00379112	0.123195	0.155013	0.00311021	0.0135886	3.4605E-07	0.780	25.977
rs143043380	A	C	-0.0488238	-0.0549162	0.010074	0.0103621	0.0103627	0.0489757	2.4618E-06	0.262	22.198
rs146872795	A	G	0.04395	-0.0203459	0.01198	0.0171231	0.00928996	0.0375962	2.2377E-06	0.588	22.382
rs148342659	T	C	0.0626818	-0.00347186	0.006294	0.0291674	0.0132022	0.0290434	2.0587E-06	0.905	22.542
rs1611579	C	T	-0.0171376	0.00647159	0.124682	0.0660829	0.00319026	0.0198439	7.8075E-08	0.744	28.857
rs17112354	A	C	0.0180135	-0.0100732	0.073079	0.072517	0.00386559	0.0188184	3.1661E-06	0.592	21.715
rs2013730	C	T	0.00946086	-0.00349234	0.552068	0.468096	0.00202257	0.0097847	2.9051E-06	0.721	21.880
rs213024	C	T	-0.011047	0.00535594	0.422475	0.439431	0.00204548	0.00986	6.6501E-08	0.587	29.167
rs2732948	T	A	0.0140062	-0.00079292	0.8232	0.824267	0.00263998	0.0128345	1.1262E-07	0.951	28.147
rs3912369	T	C	-0.0100775	0.0123215	0.322235	0.319034	0.00216227	0.0105044	3.156E-06	0.241	21.721
rs4422299	A	C	0.00930381	-0.00434391	0.497975	0.421254	0.00201598	0.0098929	3.9346E-06	0.661	21.299
rs57820851	T	G	0.0113103	0.00937744	0.349696	0.415435	0.00211044	0.0099022	8.373E-08	0.344	28.721
rs61997596	A	G	0.0125915	0.0178365	0.190789	0.169832	0.00255745	0.013045	8.5145E-07	0.172	24.240
rs62310964	C	T	0.0169474	0.0133083	0.083722	0.0844364	0.00363508	0.0176317	3.1322E-06	0.450	21.736
rs74732212	T	G	0.0314838	-0.00375849	0.022803	0.013074	0.00688653	0.0434045	4.8405E-06	0.931	20.901
rs76837702	T	C	-0.0212473	0.0137264	0.056039	0.0388983	0.00439576	0.0255067	1.3426E-06	0.590	23.364
rs78008841	T	G	-0.0248345	0.0143482	0.035287	0.0322333	0.00543126	0.0279059	4.824E-06	0.607	20.908

rs79369857	A	G	-0.0463685	-0.016256	0.0103	0.0102456	0.00993806	0.0485883	3.0783E-06	0.738	21.769
rs9460928	A	T	0.00994342	0.00109216	0.353484	0.361332	0.00210564	0.0101436	2.3349E-06	0.914	22.300
rs9472218	T	C	0.0102784	-0.0119131	0.629778	0.552005	0.00209028	0.0098893	8.7886E-07	0.228	24.179

*: To ensure an adequate number of SNPs, we employed a less stringent formula for calculating F: $F = \beta^2/se^2$.

(N) Asthma as outcome and “anxiety disorders” as exposure.

SNP	effect_allele	other_allele	β. exposure	β.outcome	eaf. exposure	eaf. outcome	se.exposure	se.outcome	*P*.exposure	*P*.outcome	F
rs1709393	T	C	-0.1509	-0.00388355	0.5793	0.4938	0.0267	0.0098162	1.651E-08	0.692	194.258
rs2146346	A	G	0.1441	-0.00910246	0.5869	0.605166	0.0298	0.009981	1.375E-06	0.362	130.364
rs2753188	A	G	0.1603	0.00203485	0.7268	0.689669	0.0332	0.0105322	1.417E-06	0.847	132.140
rs28373923	A	G	0.4193	0.0199028	0.0675	0.07216	0.0915	0.0190583	4.558E-06	0.296	197.048
rs58990403	A	G	-0.1851	-0.00206394	0.792	0.759763	0.0391	0.0115008	2.163E-06	0.858	197.610
rs739315	A	G	-0.1537	-0.0234332	0.5688	0.555282	0.0329	0.0098661	3.027E-06	0.018	180.000

(O) Asthma as outcome and “major depression disorder” as exposure.

SNP	effect_allele	other_allele	β. exposure	β.outcome	eaf. exposure	eaf. outcome	se.exposure	se.outcome	*P*.exposure	*P*.outcome	F
rs1021363	G	A	-0.03	-0.00217394	0.6434	0.725952	0.0045	0.010957	2.2872E-11	0.843	333.369
rs10235664	C	T	-0.027	-0.032538	0.2529	0.336781	0.0049	0.0103651	4.677E-08	0.002	222.400
rs10913112	T	C	-0.0262	-0.0126998	0.378	0.373795	0.0045	0.0101209	4.525E-09	0.210	260.582
rs12919291	C	G	0.0327	0.0181928	0.1884	0.154186	0.0055	0.0135451	3.092E-09	0.179	263.983
rs12967143	C	G	-0.0345	-0.00052046	0.7012	0.746183	0.0047	0.0112327	2.527E-13	0.963	402.572
rs13037326	T	C	0.031	0.0193486	0.2597	0.28248	0.0049	0.0108375	2.398E-10	0.074	298.293
rs150186873	C	A	0.0704	0.039915	0.0327	0.0177566	0.012	0.0370869	4.5131E-09	0.282	253.115
rs150346963	T	C	0.0283	0.00154355	0.4118	0.524157	0.0044	0.009832	1.157E-10	0.875	313.196
rs1931388	G	A	-0.0295	-0.0313927	0.4042	0.426541	0.0044	0.0098945	1.68E-11	0.002	338.344
rs1950829	G	A	-0.0297	-0.024509	0.5173	0.489755	0.0043	0.0097696	4.7381E-12	0.012	355.583
rs198457	T	C	-0.0315	-0.0182054	0.1886	0.101606	0.0056	0.0162894	1.9E-08	0.264	245.169
rs2111592	A	G	0.0263	0.0133748	0.3141	0.346468	0.0046	0.01026	1.35E-08	0.192	240.608
rs2214123	G	A	-0.0261	-0.0143972	0.6466	0.6224	0.0045	0.0101125	8.556E-09	0.155	251.332
rs2232423	G	A	-0.062	0.00488631	0.1056	0.0503546	0.007	0.0225635	1.135E-18	0.829	585.954
rs2418449	C	T	-0.0281	-0.0135905	0.281	0.322197	0.0048	0.0104623	4.245E-09	0.194	257.578
rs247910	G	A	0.0237	0.0021649	0.457	0.464872	0.0043	0.0097871	4.7121E-08	0.825	225.057
rs2522831	C	T	0.024	0.0265497	0.4739	0.486799	0.0043	0.0097512	2.113E-08	0.006	231.874
rs2568958	A	G	0.0382	0.0145141	0.6042	0.645171	0.0044	0.0102281	2.902E-18	0.156	563.222
rs28541419	G	C	-0.0292	-0.00390659	0.2308	0.165933	0.0052	0.0131796	1.756E-08	0.767	244.405
rs30266	A	G	0.0366	0.0317453	0.3271	0.289686	0.0046	0.0107575	1.4279E-15	0.003	475.923
rs354155	C	G	-0.0449	-0.0400846	0.0923	0.158619	0.0075	0.0134398	1.751E-09	0.003	272.703
rs3807865	A	G	0.031	0.0242838	0.4105	0.363982	0.0044	0.0101395	1.093E-12	0.017	375.421
rs4141983	C	T	-0.0264	-0.00229842	0.326	0.342228	0.0046	0.010312	9.6919E-09	0.824	247.259
rs4497414	C	T	0.0291	0.0101188	0.44	0.565788	0.0044	0.0098879	2.9269E-11	0.306	336.857
rs4799949	T	C	-0.0292	-0.0245041	0.6684	0.734523	0.0046	0.0110646	1.403E-10	0.027	305.107

rs4936276	C	G	0.0278	0.0135981	0.622	0.791466	0.0044	0.0121064	3.57E-10	0.261	293.369
rs508502	T	C	-0.0264	-0.0145577	0.2992	0.342019	0.0048	0.0103605	3.556E-08	0.160	235.959
rs66511648	C	T	0.0297	-0.00412008	0.284	0.215653	0.0048	0.0119146	6.03E-10	0.729	289.593
rs7152906	C	T	0.0258	0.0149817	0.5196	0.494129	0.0043	0.0097709	1.873E-09	0.125	268.267
rs7241572	A	G	0.0323	0.0313861	0.2047	0.216484	0.0054	0.0118543	2.433E-09	0.008	274.225
rs72948506	A	G	0.0265	0.0218011	0.2975	0.30314	0.0047	0.0106429	1.715E-08	0.041	236.972
rs7538938	C	T	0.0251	0.00632429	0.5599	0.532553	0.0043	0.0098193	7.289E-09	0.520	250.654
rs754287	A	T	-0.0289	0.00346245	0.3664	0.375997	0.0045	0.01011	1.311E-10	0.732	313.039
rs7551758	G	T	0.0283	0.0146944	0.5329	0.534819	0.0043	0.0097902	5.1074E-11	0.133	321.851
rs7725715	A	G	0.029	0.0182386	0.5343	0.614613	0.0043	0.0100399	1.6069E-11	0.069	337.836
rs843812	A	G	0.0248	0.0057277	0.4117	0.463476	0.0044	0.0097936	1.405E-08	0.559	240.521
rs9364755	G	A	0.0283	0.0062062	0.2262	0.276751	0.0051	0.0109076	3.486E-08	0.569	226.346
rs9529218	T	C	-0.034	-0.0309198	0.2031	0.281761	0.0054	0.0108776	2.231E-10	0.004	302.071
rs9536381	T	C	0.0255	0.0189502	0.3259	0.34032	0.0046	0.010304	2.617E-08	0.066	230.656
rs9831648	T	G	-0.0292	0.00760905	0.7739	0.839272	0.0052	0.013352	1.586E-08	0.569	240.891

DataTable S2 The SNPs used as genetic instruments in the Mendelian randomization (MR) analyses for the exposure of asthma, and the outcomes are "sensation of anxiety or depression", "anxiety sensation", "depression sensation", "anxiety disorders", and "major depression disorder", respectively.

(P) Asthma as exposure and "sensation of anxiety or depression" as outcome.

SNP	effect_allele	other_allele	β. exposure	β.outcome	eaf. exposure	eaf. outcome	se.exposure	se.outcome	*P*.exposure	*P*.outcome	F
rs10815391	G	T	0.0819387	-7.9185E-05	0.185765	0.231661	0.0123964	0.0011631	3.8468E-11	0.950	512.699
rs12374521	T	C	0.0600618	0.000377543	0.586507	0.544079	0.00991712	0.0009798	1.3922E-09	0.700	441.556
rs12440067	A	C	0.0626594	0.00014512	0.283883	0.245639	0.0107602	0.0011452	5.771E-09	0.900	402.789
rs12629932	G	A	0.0581266	0.000237068	0.457168	0.468915	0.00978177	0.000979	2.8099E-09	0.810	423.163
rs12950511	T	C	0.0570181	0.00144048	0.383372	0.340627	0.0100346	0.0010394	1.3299E-08	0.170	387.815
rs1837253	C	T	0.0821611	0.00271521	0.752752	0.739559	0.0116119	0.0011121	1.488E-12	0.015	634.596
rs2160203	G	A	-0.0732124	0.000379687	0.315575	0.234854	0.0105967	0.00115	4.8798E-12	0.740	584.645
rs2797409	C	T	0.058152	-0.00119138	0.320544	0.243304	0.0103854	0.0011381	2.151E-08	0.300	371.626
rs3104411	A	G	0.0568313	-0.00045749	0.614009	0.603274	0.0100819	0.0010268	1.7305E-08	0.660	386.260
rs34290285	A	G	-0.0656576	0.00217992	0.216897	0.256744	0.0119752	0.001117	4.1863E-08	0.051	369.459
rs4947324	T	C	-0.0909799	0.00406915	0.157775	0.101119	0.0135908	0.0016156	2.1677E-11	0.012	555.394
rs56062135	T	C	0.0741602	-0.00172317	0.261165	0.23619	0.0110052	0.00115	1.5988E-11	0.130	535.814
rs6894249	G	A	0.064987	-0.00098673	0.464073	0.386038	0.00974833	0.0010031	2.62E-11	0.330	530.329
rs7213668	A	G	0.0677852	-0.00105803	0.247603	0.206942	0.0112181	0.0012103	1.5176E-09	0.380	432.020
rs72836346	C	G	0.115727	0.000427066	0.095251	0.078351	0.0162895	0.0018676	1.2087E-12	0.820	582.854
rs77481341	C	T	0.0852593	0.00159041	0.1614	0.116852	0.0130503	0.0015187	6.4402E-11	0.290	496.689
rs817313	G	T	-0.058379	-0.00129967	0.615128	0.591329	0.0100629	0.0010016	6.5754E-09	0.190	407.178

(Q) Asthma as exposure and "anxiety sensation" as outcome.

SNP	effect_allele	other_allele	β. exposure	β.outcome	eaf. exposure	eaf. outcome	se.exposure	se.outcome	*P*.exposure	*P*.outcome	F
rs10815391	G	T	0.0819387	-0.00262421	0.185765	0.231647	0.0123964	0.0012225	3.8468E-11	0.032	512.699
rs12374521	T	C	0.0600618	0.00333271	0.586507	0.543859	0.00991712	0.0010298	1.3922E-09	0.001	441.556
rs12440067	A	C	0.0626594	0.000694932	0.283883	0.245446	0.0107602	0.0012037	5.771E-09	0.560	402.789
rs12629932	G	A	0.0581266	0.000193754	0.457168	0.468833	0.00978177	0.0010286	2.8099E-09	0.850	423.163
rs12950511	T	C	0.0570181	0.000347992	0.383372	0.340696	0.0100346	0.0010924	1.3299E-08	0.750	387.815
rs1837253	C	T	0.0821611	0.000374896	0.752752	0.739535	0.0116119	0.0011687	1.488E-12	0.750	634.596
rs2160203	G	A	-0.0732124	0.000818973	0.315575	0.234783	0.0105967	0.0012083	4.8798E-12	0.500	584.645
rs2797409	C	T	0.058152	0.000361984	0.320544	0.243466	0.0103854	0.0011954	2.151E-08	0.760	371.626
rs3104411	A	G	0.0568313	-0.00146571	0.614009	0.603379	0.0100819	0.001079	1.7305E-08	0.170	386.260
rs34290285	A	G	-0.0656576	0.00100624	0.216897	0.25673	0.0119752	0.0011736	4.1863E-08	0.390	369.459
rs4947324	T	C	-0.0909799	0.00204036	0.157775	0.101153	0.0135908	0.0016974	2.1677E-11	0.230	555.394
rs56062135	T	C	0.0741602	0.000169037	0.261165	0.236257	0.0110052	0.0012086	1.5988E-11	0.890	535.814
rs6894249	G	A	0.064987	0.000269006	0.464073	0.385837	0.00974833	0.0010542	2.62E-11	0.800	530.329
rs7213668	A	G	0.0677852	-0.00013198	0.247603	0.207002	0.0112181	0.0012717	1.5176E-09	0.920	432.020
rs72836346	C	G	0.115727	-0.00140583	0.095251	0.078315	0.0162895	0.0019622	1.2087E-12	0.470	582.854
rs77481341	C	T	0.0852593	0.000844649	0.1614	0.116876	0.0130503	0.0015956	6.4402E-11	0.600	496.689

rs817313	G	T	-0.058379	0.000235011	0.615128	0.591351	0.0100629	0.0010526	6.5754E-09	0.820	407.178

(R) Asthma as exposure and “depression sensation” as outcome.

SNP	effect_allele	other_allele	β. exposure	β.outcome	eaf. exposure	eaf. outcome	se.exposure	se.outcome	*P*.exposure	*P*.outcome	F
rs10815391	G	T	0.0819387	-0.00287059	0.185765	0.231217	0.0123964	0.0023924	3.8468E-11	0.230	512.699
rs12374521	T	C	0.0600618	0.000101792	0.586507	0.544494	0.00991712	0.0020141	1.3922E-09	0.960	441.556
rs12440067	A	C	0.0626594	-0.00024381	0.283883	0.244143	0.0107602	0.0023545	5.771E-09	0.918	402.789
rs12629932	G	A	0.0581266	0.00089914	0.457168	0.469925	0.00978177	0.0020125	2.8099E-09	0.655	423.163
rs12950511	T	C	0.0570181	0.00156481	0.383372	0.34253	0.0100346	0.0021379	1.3299E-08	0.464	387.815
rs1837253	C	T	0.0821611	0.0014322	0.752752	0.74092	0.0116119	0.0022896	1.488E-12	0.532	634.596
rs2160203	G	A	-0.0732124	-0.00323493	0.315575	0.233312	0.0105967	0.0023697	4.8798E-12	0.172	584.645
rs2797409	C	T	0.058152	0.000700984	0.320544	0.243247	0.0103854	0.0023428	2.151E-08	0.765	371.626
rs3104411	A	G	0.0568313	-0.00325169	0.614009	0.604427	0.0100819	0.002116	1.7305E-08	0.124	386.260
rs34290285	A	G	-0.0656576	-0.00379845	0.216897	0.256726	0.0119752	0.0023027	4.1863E-08	0.099	369.459
rs4947324	T	C	-0.0909799	-0.00226667	0.157775	0.101004	0.0135908	0.0033331	2.1677E-11	0.496	555.394
rs56062135	T	C	0.0741602	-0.00214499	0.261165	0.236304	0.0110052	0.0023679	1.5988E-11	0.365	535.814
rs6894249	G	A	0.064987	-0.00443504	0.464073	0.386947	0.00974833	0.0020648	2.62E-11	0.032	530.329
rs7213668	A	G	0.0677852	-0.00151413	0.247603	0.207923	0.0112181	0.0024834	1.5176E-09	0.542	432.020
rs72836346	C	G	0.115727	-0.00507275	0.095251	0.078336	0.0162895	0.0038476	1.2087E-12	0.187	582.854

rs77481341	C	T	0.0852593	-0.00197129	0.1614	0.116573	0.0130503	0.0031273	6.4402E-11	0.528	496.689
rs817313	G	T	-0.058379	0.000203704	0.615128	0.593115	0.0100629	0.0020658	6.5754E-09	0.921	407.178
rs9272042	C	A	-0.0909111	-0.00181082	0.602012	0.492079	0.0100021	0.002013	9.9747E-20	0.368	1001.659

(S) Asthma as exposure and "anxiety disorders" as outcome.

SNP	effect_allele	other_allele	β. exposure	β.outcome	eaf. exposure	eaf. outcome	se.exposure	se.outcome	*P*.exposure	*P*.outcome	F
rs10815391	G	T	0.0819387	0.0518	0.185765	0.2084	0.0123964	0.0346	3.8468E-11	0.134	512.699
rs12374521	T	C	0.0600618	0.0392	0.586507	0.5373	0.00991712	0.0304	1.3922E-09	0.197	441.556
rs12440067	A	C	0.0626594	-0.0102	0.283883	0.2206	0.0107602	0.0342	5.771E-09	0.766	402.789
rs12629932	G	A	0.0581266	0.0125	0.457168	0.4644	0.00978177	0.0263	2.8099E-09	0.634	423.163
rs12950511	T	C	0.0570181	-0.0257	0.383372	0.3513	0.0100346	0.0297	1.3299E-08	0.387	387.815
rs1837253	C	T	0.0821611	-0.0028	0.752752	0.7326	0.0116119	0.0339	1.488E-12	0.935	634.596
rs2160203	G	A	-0.0732124	-0.0457	0.315575	0.2364	0.0105967	0.0311	4.8798E-12	0.142	584.645
rs2797409	C	T	0.058152	-0.0169	0.320544	0.237	0.0103854	0.0308	2.151E-08	0.584	371.626
rs4947324	T	C	-0.0909799	0.0205	0.157775	0.105	0.0135908	0.0502	2.1677E-11	0.683	555.394
rs56062135	T	C	0.0741602	-0.052	0.261165	0.234	0.0110052	0.0351	1.5988E-11	0.138	535.814
rs6894249	G	A	0.064987	0.0171	0.464073	0.3826	0.00974833	0.0273	2.62E-11	0.530	530.329
rs72836346	C	G	0.115727	0.0144	0.095251	0.0857	0.0162895	0.0586	1.2087E-12	0.806	582.854
rs77481341	C	T	0.0852593	-0.0183	0.1614	0.1095	0.0130503	0.0468	6.4402E-11	0.696	496.689
rs817313	G	T	-0.058379	-0.0385	0.615128	0.5751	0.0100629	0.0392	6.5754E-09	0.326	407.178

(T) Asthma as exposure and "major depression disorder" as outcome.

SNP	effect_allele	other_allele	β. exposure	β.outcome	eaf. exposure	eaf. outcome	se.exposure	se.outcome	*P*.exposure	*P*.outcome	F
rs10815391	G	T	0.0819387	-0.0041	0.185765	0.2276	0.0123964	0.0052	3.8468E-11	0.424	510.618
rs12374521	T	C	0.0600618	0.0023	0.586507	0.5447	0.00991712	0.0043	1.3922E-09	0.588	440.012
rs12440067	A	C	0.0626594	0.0053	0.283883	0.2423	0.0107602	0.0051	5.771E-09	0.301	401.504
rs12629932	G	A	0.0581266	-0.0003	0.457168	0.4703	0.00978177	0.0043	2.8099E-09	0.940	421.745
rs12950511	T	C	0.0570181	0.0089	0.383372	0.3416	0.0100346	0.0046	1.3299E-08	0.053	386.624
rs1837253	C	T	0.0821611	0.0081	0.752752	0.7379	0.0116119	0.0049	1.488E-12	0.099	631.411
rs2160203	G	A	-0.0732124	0.0008	0.315575	0.2359	0.0105967	0.0051	4.8798E-12	0.881	581.940
rs2797409	C	T	0.058152	0.0003	0.320544	0.2426	0.0103854	0.005	2.151E-08	0.945	370.532
rs56062135	T	C	0.0741602	-0.0047	0.261165	0.2362	0.0110052	0.0051	1.5988E-11	0.356	533.542
rs6894249	G	A	0.064987	-0.0076	0.464073	0.3859	0.00974833	0.0044	2.62E-11	0.083	528.104
rs7213668	A	G	0.0677852	0.0045	0.247603	0.2078	0.0112181	0.0053	1.5176E-09	0.395	430.543
rs77481341	C	T	0.0852593	0.0125	0.1614	0.1163	0.0130503	0.0067	6.4402E-11	0.061	494.737
rs817313	G	T	-0.058379	-0.003	0.615128	0.5872	0.0100629	0.0045	6.5754E-09	0.504	405.865
